# An Improved Phenotyping Method for Evaluation of Yam (*Dioscorea* spp.) Resistance to Nematodes Belonging to the Genera *Meloidogyne* and *Scutellonema*

**DOI:** 10.3390/plants13091175

**Published:** 2024-04-23

**Authors:** Yao A. Kolombia, P. Lava Kumar, Antonio J. Lopez-Montes, Abiodun O. Claudius-Cole, Norbert G. Maroya, Nicole Viaene, Wim Bert, Danny L. Coyne

**Affiliations:** 1 International Institute of Tropical Agriculture (IITA), PMB 5320, Oyo Road, Ibadan P.O. Box 200001, Nigeria; l.kumar@cgiar.org (P.L.K.); mijuamarel@gmail.com (A.J.L.-M.); bi_cole@yahoo.com (A.O.C.-C.); norbertmaroya2014@gmail.com (N.G.M.); D.COYNE@CGIARG.ORG (D.L.C.); 2 Nematology Research Unit, Department of Biology, Ghent University, K.L. Ledeganckstraat 35, 9000 Gent, Belgium; nicole.viaene@ilvo.vlaanderen.be (N.V.); wim.bert@ugent.be (W.B.); 3 Department of Crop Protection and Environmental Biology, University of Ibadan, Ibadan P.O. Box 200132, Nigeria; 4 Flanders Research Institute for Agriculture, Fisheries and Food (ILVO), Burgemeester Van Gansberghelaan 96, 9820 Merelbeke, Belgium; 5 IITA, Kasarani, Nairobi P.O. Box 30772-00100, Kenya

**Keywords:** phenotyping, host resistance, root-knot nematode, *Scutellonema bradys*, yam, West Africa

## Abstract

Phenotyping yam (*Dioscorea* spp.) germplasm for resistance to parasitic nematodes is hampered by the lack of an efficient screening method. In this study, we developed a new method using rooted yam vine cuttings and yam plantlets generated from semi-autotrophic hydroponics (SAHs) propagation for phenotyping yam genotypes for nematode resistance. The method was evaluated using 26 genotypes of *D. rotundata* for their reaction to *Scutellonema bradys* and four root-knot nematode species, *Meloidogyne arenaria*, *M. enterolobii*, *M. incognita*, and *M. javanica*. Yam plantlets established in nursery bags filled with steam-sterilized soil were used for screening against single nematode species. Plants were inoculated four weeks after planting and assessed for nematode damage eight weeks later. A severity rating scale was used to classify genotypes as resistant, tolerant, or susceptible determine based on the nematode feeding damage on tubers and the rate of nematode multiplication in the roots of inoculated plants. The results demonstrated putative resistance and tolerance against *S. bradys* in 58% of the genotypes and 88%, 65%, 65%, and 58% against *M. arenaria*, *M. javanica*, *M. incognita*, and *M. enterolobii*, respectively. The method is rapid, flexible, and seasonally independent, permitting year-round screening under controlled conditions. This method increases the throughput and speed of phenotyping and improves the selection process.

## 1. Introduction

Yam, a member of Dioscoreaceae, serves as a staple food in West Africa, supplying the daily carbohydrate needs of more than 350 million individuals [1,2,3]. The most commonly grown edible yam in this region is the white yam (*D. rotundata* Poir.) of the genus *Dioscorea* [4,5]. However, yam cultivation faces significant challenges due to nematodes [6], particularly the yam nematode *Scutellonema bradys*, as well as root-lesion nematodes (RLNs) (*Pratylenchus* genus) and root-knot nematodes (RKNs) (*Meloidogyne* genus), which are critical threats to both yam yields and the quality of the tubers in West Africa [7,8,9].

Nematode infection and feeding can result in symptoms of dry rot, characterized by cracked or flaky surfaces (in the case of RLN infestation) or tuber deformation with galls and so-called crazy roots (in the case of RKN infestation), resulting in a deterioration of tubers and production losses [7]. Although the synthetic pesticides can minimize nematode infection, the practice is unusual due to limited access, unaffordability, and the small market supply as most noxious nematicides are strictly regulated [10,11,12]. In addition, nematicide application is discouraged due to environmental and food safety concerns. Moreover, the local agricultural practice of mixed cropping may lead to the accumulation of nematode populations and possibly the abandonment of badly afflicted fields [13].

Using resistant cultivars is one of the most effective techniques to control the nematodes [14]. However, the development of resistant yam varieties has progressed slowly due to the absence of precise and effective screening methods and the challenge of producing adequate planting material for continuous phenotyping throughout the year. Conventionally, yam cultivation utilizes whole tubers or tuber segments as planting materials [15]. However, inherent tuber dormancy (up to 5 months after harvest) makes it difficult to conduct routine year-round screening. The length of dormancy depends on the date of harvest, species, cultivar, and environmental conditions during storage [16,17].

The limitations of traditional yam screening, which relies on using tubers as planting materials are (i) the natural break in the evaluation cycle each year due to tuber dormancy [4,17]; (ii) the lack of healthy planting material [7]; (iii) the season, as the dry season poses difficulties in conducting field trials; (iv) the extended period of the yam’s growth phase in the field; (v) difficulty in controlled inoculation under field conditions; (vi) and, most importantly, difficulties in waiting to assess nematode multiplication in the roots or damage to tubers due to nematode feeding until after harvest. Furthermore, challenges relating to using different scoring scales in different studies and differences in the classification of plant reaction to nematode feeding and reproduction rate [14,18,19] remain issues that need to be addressed to standardize and have the same interpretation in yam phenotyping. Accordingly, standardization of damage scoring scales and plant reaction classification is required to establish a reference frame for screening yam germplasm.

To address the issue of nematode-free planting materials, the International Institute of Tropical Agriculture (IITA) developed novel propagation methods for yam that include vine cuttings (VCs) and aeroponics [15,20] and semi-autotrophic hydroponics (SAHs). While the VC (one or two nodal cuts) planting materials are generally prepared from vines deriving from (1) minisetts tubers planted in pots or (2) aeroponics and rooted in substrates in nursery bags, the SAH planting materials are nodal cuts from tissue culture (TC) that are planted and rooted in compost peat materials or cocopeat media that enable a faster growth.

In this study, we used rooted vines generated from conventional vine cuttings and SAH propagation for rapid evaluation of a select set of yam genotypes against four species of RKN (*M. arenaria*, *M. enterolobii*, *M. incognita,* and *M. javanica*) and the yam nematode (*Scutellonema bradys*). We further reviewed and addressed the challenges relating to two commonly used plant reaction classification systems based on nematode multiplication rate in the roots and feeding damage to tubers [14,18]. We discussed the benefits of two new yam planting materials that offer convenience for year-round testing of genotype responses to specific nematode species under screenhouse conditions.

## 2. Results

### 2.1. Planting Material Survival Rate before Nematode Inoculation

Survival of planting material for at least three months is an important consideration for nematode phenotyping. The rooted vine cuttings of all genotypes generated through conventional vine cutting methods, one week after planting (WAP), exhibited a survival rate above 50% (Supplementary Figure S1). At two and three WAPs, the survival rate of breeder’s lines and cultivar decreased. Only three genotypes at four WAPs had a survival rate above 50%, with TDr 96/01817 (80%) and TDr 00/00403 (70%) surviving the best. TDr 03/00180 did not survive four WAPs (Supplementary Figure S1A). At two and three WAPs, vine survival rates for all landraces exceeded 50% (Supplementary Figure S1B). At four WAPs, only TDr 04-219 (Amula) achieved a 57% survival rate. The landrace TDr Pouna had a 47% survival rate, whereas TDr Alumako and TDr Dente had the lowest survival rates, at 28% and 13%, respectively (Supplementary Figure S1B). However, the survival rate was >95% for genotypes (TDr Makakusa and TDr 95/19177) generated by SAH propagation at five WAPs (Supplementary Figure S2).

### 2.2. Screening Assay and Phenotyping

**Figure 1:**
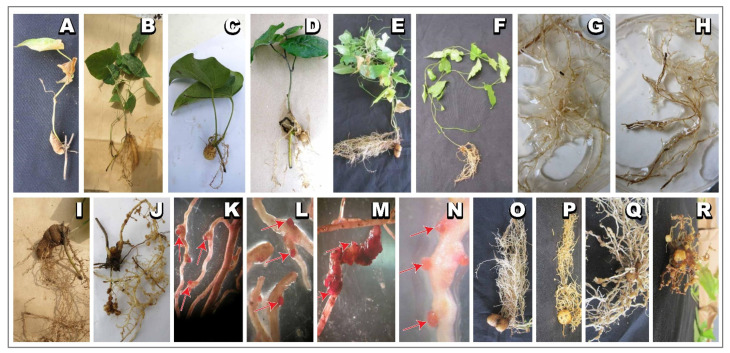
Output of yam screening for resistance to nematodes (root-knot nematodes and the *Scutellonema bradys*) using vine cuttings and SAH plantlets. Plants and roots produced by conventional vine cuttings in nursery bags (**A**–**D**,**F**–**N**). Plants generated from vine cuttings (**A**–**D**,**F**), roots without damages (**G**), roots with necrosis (**H**), galled root system (**I**,**J**) with stained egg masses (red arrow) (**K**–**N**). Plants generated from SAHs (**E**), root systems (**O**,**P**), galls and crazy roots on yam tubers (**Q**,**R**) due to *Meloidogyne incognita* inoculated 12 weeks after planting and evaluated 8 weeks after inoculation.

**Figure 2:**
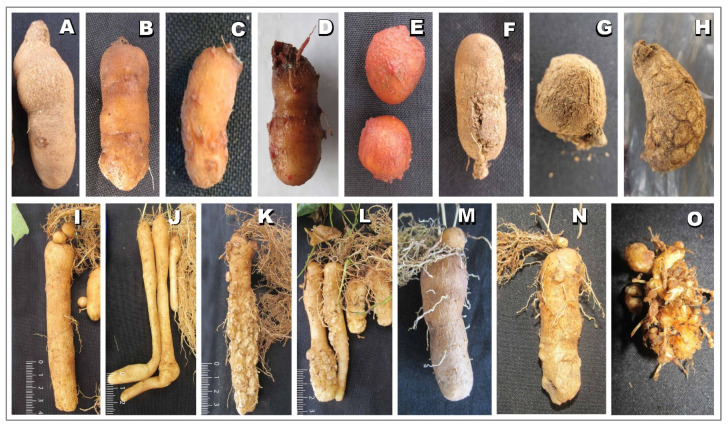
Output of yam screening for resistance to nematodes (root-knot nematodes and the *Scutellonema bradys*) using vine cuttings and SAH plantlets. Tubers produced by conventional vine cuttings in nursery bags (**A**–**H**). Tuber without symptoms (**A**), tuber with galls (**B**–**E**), tuber with cracks and dry rot (**F**–**H**) eight weeks after inoculation. Tubers generated by vine cuttings in 3 L pots (**I**–**L**). Yam tubers from control (**I**,**J**), galls and crazy roots on yam tubers due to *Meloidogyne incognita* (**K**,**L**) inoculated 12 weeks after planting and evaluated eight weeks after inoculation. Plants generated from SAHs in 3 L pots (**M**–**O**). Yam tuber from control (**M**), galls and crazy roots on yam tubers (**N**,**O**) due to *Meloidogyne incognita* inoculated 12 weeks after planting and evaluated 8 weeks after inoculation.

The genotype reaction to nematodes is summarized in Table 1. Generally, no apparent differences in the above ground shoot growth of inoculated and control plants were observed. At the time of harvesting, 8 weeks post-inoculation, in both control and nematode-inoculated plants, plants had well-established root systems and small yam tubers (Figure 1 and Supplementary Tables S1–S5). Plants generated from SAHs had more dense root growth (Figure 1E) compared with plants from conventional vine cuttings (Figure 1F). The average tuber weight in the uninoculated control group ranged from 0.02 g to 3.22 g, with the majority of accessions weighing less than 1 g (Supplementary Tables S1–S5; Supplementary Figure S3). Tuber formation was 100% in some genotypes, and 50 to 70% of the plants produced tubers. In the inoculated plants (Figure 1 and Figure 2), RKN-inoculated plants showed galls on their tubers and roots (Figure 1I–R), and there were cracks on tubers and necrosis on roots on plants inoculated with *S. bradys* (Figure 2F–H). For vine-cutting performance evaluation, large tubers, galls, and “crazy roots” were observed when 3 L pots were used compared to observation in 1 L nursery bag (Figure 2I–L). A similar observation was made when SAH materials were used in the 3 L pots (Figure 2M–O).

Based on host designation after Sasser et al. [18], 65% of the 26 genotypes evaluated were classified as resistant (R) to *M. incognita*, including the released cultivar, 11 breeder’s lines, and 5 landraces (Table 1). A total of 65% of genotypes were resistant to *M. javanica*, including the released cultivar, 13 breeder’s lines, and 3 landraces (Table 1). For *M. enterolobii*, 58% of the genotypes were resistant: 10 breeder’s lines and 5 landraces (TDr Agbanwobe, TDr Dente, TDr Makakusa, TDr 04-219 (Amula), and TDr 08-21-2 (Ekpe)) (Table 1). Following *M. arenaria* inoculation, 88% of genotypes, including the released cultivar, 18 breeder’s lines, and 5 landraces were resistant (Table 1). Inoculation with *S. bradys* indicated that 62% of genotypes were resistant, including the released cultivar, 11 breeder’s lines, and 4 landraces (Table 1). Specifically, some genotypes were identified as hypersusceptible (HS) to *M. incognita* (TDr 97/00840, TDr 02/00515, TDr 00/00403, TDr 07/00168, TDr 95/18544, TDr 98/00933, TDr 03/00058, TDr Makakusa, TDr Bp122 (TDr Pouna)); *M. javanica* (TDr 00/00403, TDr 07/00168, TDr 95/18544, TDr 98/00933, TDr 01/00405, TDr 08-21-2 (Ekpe), TDr Alumako, TDr 2341 (Amula), TDr Bp122 (TDr Pouna)); *M. enterolobii* (TDr 94/01108, TDr 95/18544, TDr 03/00058); *M. arenaria* (TDr Agbanwobe); and *S. bradys* (TDr 00/00362, TDr 03/00180, TDr 89/02672, TDr 97/00840, TDr 95/18544, TDr 03/00058, TDr 2341 (Amula), TDr Makakusa) (Table 1). Based on the host designation after Starr et al. [14], all the genotypes categorized as resistant by Sasser et al., 1984 [18] were “resistant and tolerant” (Table 1), while the hypersusceptible genotypes were “resistant and intolerant” (Table 1). In the present study, evaluation of five genotypes that have previously been tested (reference materials/checks) [21,22,23,24] revealed similar conclusion for three genotypes (i.e. susceptible and hypersusceptible), but contrasting results observed for two genotypes, as the current evaluation predicted genotypes as resistant but previous studies predicted those as susceptible (Table 2).

In general, among the five breeder’s lines (TDr 03/00196, TDr 95/01932, TDr 99/02674, TDr 96/01817, and TDr 07/00873), one improved and released variety (TDr 89/02475) and one landrace (TDr Dente) were identified to be resistant to the tested RKNs and *S. bradys* (Table 1).

The SAH-derived materials produced significantly heavier tubers (*p* = 0.0109) and roots (*p* = 0.0027) than plants established from vine cuttings (Table 3). For instance, the root weight of TDr Makakusa generated in SAHs (5.5 g) was averagely higher (*p* = 0.0027), compared to plants generated by VCs (0.04 g). The observed trend was maintained even after transplanting plants from nursery bags to larger pots, suggesting better performance of the plants generated from SAHs compared to VCs. The nematode reproduction factor was significantly higher (*p* = 0.0002 and *p* = 0.0655) in plants produced under SAH conditions compared with VCs (Table 4), enabling clear discrimination between resistant and susceptible genotypes. The reason for this was the well-established root system.

## 3. Discussion

Optimal pest and disease control in yam cultivation can be achieved using stable plant resistance, which is easy to adopt and cost-efficient [25]. Identifying and applying resistance against nematodes is one of the goals of the yam breeding programs of IITA and several national programs in West Africa, where *S. bradys* and *Meloidogyne* spp. are prevalent and considered major yam pests [7,9,24]. To date, few nematode resistance sources have been reported [7,9,24,26,27,28]. Difficulties in establishing optimal conditions (nematode-free planting materials, controlled environments, and accurately recognized nematode species) for screening germplasm has been one of the major challenges for nematode resistance breeding.

Yams are traditionally propagated using tubers which may carry nematodes and diseases that influence the screening output. Moreover, generating plants from setts is time consuming and results in non-uniform growth due to irregular emergence rates. Rooted VC and SAH plantlets derived from pest- and disease-free stocks provide uniform planting material that can be readily propagated and raised in screenhouses under pest-free conditions. Compared with VCs, SAH plantlets are better for phenotyping owing to a better growth rate, rooting, and ability to produce tubers, and provide data for both roots (nematode multiplication rate) and tubers (damage severity). 

The results of the reference samples (check genotypes) showed that screening yam using VCs is a reliable method, as resistant genotypes were observed to be resistant to inoculated nematodes. In contrast, susceptible materials were found to be hypersusceptible to most of the inoculated nematode species. Furthermore, we found that genotype reactions to nematodes are better expressed by the designations proposed Sasser et al. [18] than by those of Starr et al. [14]. Accordingly, we believe that the classification of host-plant reactions proposed by Sasser et al. [18] would be preferable for phenotyping.

In the present study, the procedure for phenotyping yam germplasm for resistance to nematodes was refined using yam VCs. Conventional screening methods involve using yam tubers, cut tuber setts (minisetts), and pots with untreated soil or field plots. However, the use of tubers or minisetts as planting materials [29,30] is constrained to varying extents by cost and space as well as to a large degree by the inability to maintain screening activities year-round due to inherent dormancy [15,31]. Moreover, using these materials is often associated with a number of confounding factors, including sprouting time, rooting, and above-ground biomass produced from the section of the tuber from which the minisett is derived, thereby potentially complicating the interpretation of results. Furthermore, field trials may result in other nematode infections [32] or infections with viruses, which often only become evident during vegetative growth. In addition, it is difficult to specifically screen for a particular RKN species using conventional screening under natural field conditions [33]. However, the results obtained in the present study highlight the advantage of screening genotypes separately against each nematode species to determine precise responses. In this regard, it is well established that, despite having a wide host range, RKN species may exhibit varying degrees of specificity toward different host cultivars [34]. For example, it has been observed that cultivars tolerant to the tropical species *M. arenaria*, *M. incognita*, and *M. javanica* were susceptible to *M. enterolobii* [35]. Thus, it is necessary to conduct separate assessments for each nematode species to identify those genotypes with multiple resistances in the field. In addition, further evaluations should be conducted to assess the resistance against these individual species when plants are subjected to attack by multiple species [36]. While results in the present study corroborate observations of previous studies [21,22,24,37], we strongly recommend the use of most parents/landraces used in the previous studies during the protocol validation stage.

In the present study, use of VCs not only provide nematode-free planting material but are also readily obtainable from a few tubers and can be used throughout the year for successive trials using same parent stocks. In addition, the use of VCs offers relatively homogeneous planting materials [38]. However, the use of VCs does have certain limitations, among which one of the key issues observed was inadequate root development in certain genotypes (Figure 1A,C). This seems to be linked to volume of soil containers used for planting. For instance, better rooting was observed in pots which had large volume, compared to nursery bags which has small volume. This also reflected in terms of nematode multiplication. The nematode feeding damage was more pronounced in tubers derived from nursery bags likely because of high density of nematodes (Figure 2K,L). 

The method using VCs has been tested and demonstrated as a practical and reliable phenotyping method for evaluating the resistance of yam genotypes to nematodes. Compared to conventional screening procedures, using vines as screening material is less time-consuming (5 months versus 8 to 12 months) and offers greater flexibility in terms of container choice, space utilization, and planting material homogeneity. However, low survival rate of VCs and restricted root system development in some genotypes is a limitation. Additional research is necessary to improve VC survival rates, potentially through the application of rooting hormones [39,40]. Selecting vines from the same section of the plant, pre-rooting, and then selecting only the vines with sufficient root mass for phenotyping is an alternative approach to ensure homogeneity of the plants for nematode phenotyping. The limitations associated with VCs can be overcome using SAH plantlets as planting material that shows efficient rooting and tuber formation.

The comparatively low amount of dry rot observed because of the limited development of tubers highlights the necessity of assessing the reproduction of *S. bradys* in genotype evaluations, whereas the presence of galls may be sufficient for a rapid evaluation of genotype reactions to RKN species. Hence, VCs may be suitable for screening large germplasm collections. However, the use of cuttings in nursey bags (1 L) require technical skills to evaluate galls and egg masses under a stereoscopic microscope) for predicting genotype response, whereas, evaluation based on the dry rot symptoms on tubers offer simple means for non-specialists to evaluate genotype response.

During the development of this novel phenotyping approach, we identified several promising sources of resistance (TDr 03/00196, TDr 00/00362, TDr 95/01932, TDr 94/01108, TDr 03/00180, TDr 96/00604, TDr 99/02674, TDr 89/02475, TDr 89/02672, TDr 96/01817, TDr Dente, and TDr Agbanwobe) against the most prevalent *Meloidogyne* species (*M. javanica* and *M. incognita*) and *S. bradys*. It is worth testing these genotypes further in field conditions to validate host resistance.

## 4. Materials and Methods

The experiment was carried out in the screenhouse at the International Institute of Tropical Agriculture (IITA), Ibadan, Nigeria, located at latitude 7°3′ N and longitude 3°45′ E in the “derived Savannah” agro-ecological zone.

### 4.1. Yam Cultivars and Planting Material

**Figure 3:**
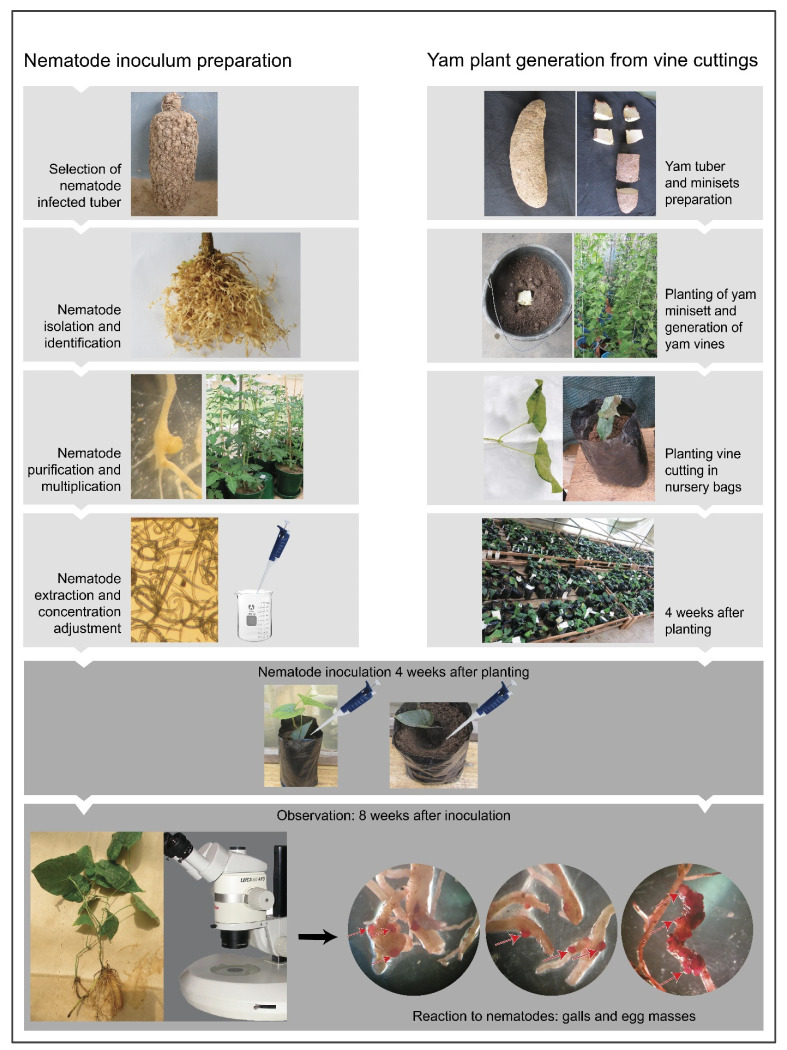
Scheme of the methodology for screening yam genotypes against nematodes (root-knot nematodes) using vine cuttings**.** Same procedure was used to screen yam genotypes again with *Scutellonema bradys*.

**Figure 4:**
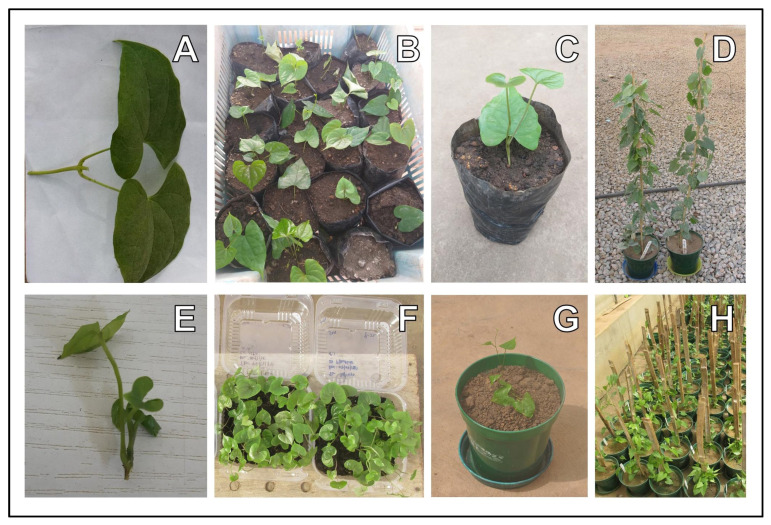
Screening yam for resistance to nematodes using rooted vine-generated planting materials: rooted vine cuttings established from vine cuttings (**A**–**D**) and plantlets generated from semi-autotrophic hydroponics (SAHs) (**E**–**H**). Vine cutting for planting (**A**); planted and acclimatized vine cuttings in 1 L nursery bags (**B**); established vine cutting ready for nematode inoculation (**C**); vine cuttings transplanted in 3 L pots (**D**). SAH planting material (**E**); established SAHs ready for planting (**F**); SAH material transplanted in 3 L pots (**G**); SAH materials inoculated with nematodes (*Meloidogyne incognita* and *M. javanica*) (**H**).

Twenty-six genotypes of white yam (*Dioscorea rotundata*) underwent evaluation. These genotypes consisted of seven landraces, one released cultivar, and 18 breeding lines developed by the IITA (International Institute of Tropical Agriculture). Among the selected genotypes for screening, three landraces (TDr 04-219 (Amula), TDr Makakusa, and TDr Pouna) one breeder’s variety (TDr 95/18544), and the released improved variety (TDr 89/02475) known to be susceptible to RKN and *S. bradys* [9,21,22,24] were used as reference samples to evaluate yam genotypes’ reaction to nematodes using plants generated from improved propagation methods based on vine cuttings and growing with SAHs. Plants generated from vine propagation or SAHs were used as source plants (Figure 3 and Figure 4).

Establishment of vine cuttings: Minisetts (20–50 g sliced tubers) of 26 yam genotypes underwent pre-treatment. They were immersed in a suspension containing a mixture of the insecticide chlorpyrifos (7 mL/L) and the fungicide mancozeb (10 g/L) for 5 min. Afterward, they were air dried for 24 h before being planted in 10 L plastic pots filled with 8 L of steam-sterilized topsoil and sand (2:1). After yam seeds sprouted, the vines were supported with stakes and tended to in the screenhouse, and they received daily watering until they were fully established. Three months after germination, the vines were cut from the third node outward from the base, and single-node VCs bearing two leaves were planted in nursery bags (1 L) or plastic pots (3 L) filled with steam-sterilized soil (soil and sand in a 2:1 ratio), as described above, and allowed to stand under screenhouse conditions for 4 weeks for rooting and establishment (Figure 2 and Figure 3). Established plants were staked whenever necessary and used for nematode inoculation after 4 weeks (Figure 3). Planting material of the genotype TDr 01/00405 was derived from the vines of a pre-established plant.

Plantlets from SAH propagation: SAHs is a new propagation method adopted for the rapid propagation of tissue culture plantlets under laboratory conditions [20,41,42] (Figure 4E–H). Yam plantlets of two genotypes, TDr Makakusa and TDr 95/19177, generated using the SAHs propagation system, were transferred to 3 L plastic pots filled with steam-sterilized topsoil and sand (2:1). Plants were staked and used for nematode inoculation after 4 weeks (Figure 4H).

### 4.2. Nematode Multiplication, Identification, and Inoculum Preparation

Various root-knot nematode (RKN) species, originating from individual egg masses found on yams, were cultivated on tomato (*Solanum lycopersicum*) cv. Marmande seedlings and plumed cockscomb (*Celosia argentea*) in 1 L pots filled with steam-sterilized soil within a screenhouse maintained at temperatures between 24 °C and 32 °C. Root-knot nematode species, including *M. arenaria*, *M. enterolobii*, *M. incognita*, and *M. javanica*, were confirmed through isozyme analysis of ten young egg-laying females and DNA sequence analysis [9,43,44]. For *S. bradys*, nematodes were extracted from the chopped peel of nematode-infested yam tubers showing symptoms of dry rot [35] using a modified Baermann method, concentrated in an aqueous suspension, and used for inoculum preparation. The nematode *S. bradys* was identified using combined morphological and molecular data [35].

### 4.3. Screening Assay and Phenotyping

Test plants generated via conventional vine propagation or SAHs were used for nematode inoculation 4 weeks after transplantation. To establish favorable conditions for nematodes, inoculated plants were watered on the day after inoculation. During the trial, plants were generally watered at 2-day intervals, although soil conditions were regularly monitored to avoid wilting or waterlogging. Inoculation was performed by introducing a suspension of nematodes in a ring made around the plant base up to the root surface. Plants of each genotype were inoculated separately in the rhizosphere zone with the four RKN species (1000 juveniles/bag and 3000 juveniles/pots to achieve a density of 1 nematode mL^−1^ soil) and *S. bradys* (500 nematodes/bag)*,* with seven replicates being used for each of the six treatments (control, *M. arenaria*, *M. enterolobii*, *M. incognita*, *M. javanica,* and *S. bradys*). Each nursery bag/pot containing a single plant (from a single node vine cutting piece) was considered a replicate. Second-stage juveniles (J2), the infective stage of *Meloidogyne* species [41], were used for RKN inoculation, whereas a mixture of juveniles, females, and males was used for *S. bradys* inoculation. Vines planted in nematode-free soil were used as controls. The experiment followed a completely randomized design, with each nematode species inoculated into all yam genotypes. Each genotype was maintained on a separate table. After eight weeks of inoculation, the experiment was terminated, and plants were assessed for their reactions to nematodes by evaluating the sensitivity of roots and tubers to nematode feeding (measured in terms of a damage index, di) and host conduciveness to support nematode multiplication (measured as host efficiency), as detailed below (Figure 3).

Each test plant’s root system was cleaned, weighed, and chopped into 5 cm portions for examination in a Petri dish under a stereoscopic microscope (Leica Model MZ9.5; magnification ×10–20) to assess galling severity and necrosis rate. Egg masses were stained with eosin B (0.1 g L^−1^ water), and the number of galls with egg masses was counted [42]. For RKN, we used a damage index based on a severity rating scale from 1 to 5: 1 = no galls, 2 = 1–2 galls, 3 = 3–10 galls, 4 = 11–30 galls, and 5 = >30 galls per plant (adapted from Sasser et al., 1984 [18]). The host sensitivity to *S. bradys* feeding was estimated based on the di measured as the percentage of the root system showing necrosis and tubers showing necrosis, dry rot, and/or cracks based on a severity rating scale of 1 to 5: 1 = no symptoms, 2 = slight damage (1%–25% of tubers and root system with symptoms), 3 = mild damage (26%–50% symptoms), 4 = heavy damage (51%–75% symptoms), and 5 = severe damage (>75% symptoms) [21,43]. The host efficiency for nematode multiplication was determined based on the nematode reproduction factor (Rf) estimated using the formula [Rf = final nematode population (Pf)/initial nematode population (Pi)], from nematodes in the soil, roots, and tubers [24,44].

### 4.4. Data Collection and Analysis

Median values of the plant damage index and means of the nematode RF were used to categorize yam genotypes. Data related to tuber and root weight, as well as the final nematode density (in soil, roots, and tubers), were collected and analyzed using a generalized linear procedure with SAS PROC GLM (SAS Institute, Cary, NC, USA), and means were compared for significance (*p* ≤ 0.05) using the least significant difference (LSD) test with SAS 9.3.

Designations of host-plant reaction to nematodes were based on host efficiency “HEf” (the ability of nematode to reproduce: nematode RF) and host sensitivity “HSe” (damage index: galling index for RKN) [23]. The host designation proposed by Sasser et al. [18] combined both HEf and HSe to classify host-plant genotypes as resistant (HEf ≤ 1 and HSe ≤ 2), tolerant (HEf > 1 and HSe ≤ 2), hypersusceptible (HEf ≤ 1 and HSe > 2), or susceptible (HEf > 1 and HSe > 2), rating damage on a scale ranging from 0 to 5. In a more recent version of host designation, Starr et al. [14] proposed the following categories: resistant and tolerant “RT” (HEf ≤ 1 and HSe ≤ 2), resistant but intolerant “RI” (HEf ≤ 1 and HSe > 2), susceptible but tolerant “ST” (HEf > 1 and HSe ≤ 2), and susceptible and intolerant “SI” (HEf > 1 and HSe > 2) (See Table 5). Hence, the term resistance (*sensu* Starr et al., 2013 [14]) with respect to the host plant refers strictly to the suppressive effect of the plant on the ability of nematodes to reproduce, whereas tolerance describes the degree of damage caused by the nematode to the host plant [14,45].

In this study, genotype reactions were characterized by combining di and Rf following the host designations proposed by Sasser et al. [18] and Starr et al. [14] based on host efficiency and host sensitivity (Table 5) in order to compare and discuss the observations obtained in the present study with those obtained in previous studies, particularly to the selected reference materials. The host designations were later discussed.

## 5. Conclusions

Screening yam germplasm for resistance to plant-parasitic nematodes has been a challenge. The present study established a new and efficient yam phenotyping protocol using alternative planting materials. It is a rapid, flexible, and seasonally independent method that permits year-round screening under controlled conditions. We have demonstrated the value of VCs and SAHs for screening yam germplasm for resistance to nematodes. We strongly recommend using SAH planting materials for screening as they produce higher root and tuber weights, hence enabling a clear and physical screening that allows evaluation of nematode damage. The method appears to stand alone for RKN screening while a post-harvest period will be required to effectively evaluate *S. bradys* damage. Validation of the putative resistance sources identified in this study under field conditions is necessary.

## Figures and Tables

**Table 1:** Classification of yam genotype response to *Meloidogyne arenaria*, *M. enterolobii*, *M. incognita*, and *M. javanica* and *Scutellonema bradys* in nursery bags after Sasser et al., 1984 (A) [18] and Starr et al., 2013 (B) [14].

N	Accession *	*M. incognita*		*M. javanica*		*M. enterolobii*		*M. arenaria*		*S. bradys*	
		A	B	A	B	A	B	A	B	A	B
1	TDr 03/00196 ^a^	R	RT	R	RT	R	RT	R	RT	R	RT
2	TDr 00/00362 ^a^	R	RT	R	RT	R	RT	R	RT	HS	RI
3	TDr 95/01932 ^a^	R	RT	R	RT	R	RT	R	RT	R	RT
4	TDr 94/01108 ^a^	R	RT	R	RT	HS	RI	R	RT	na	na
5	TDr 03/00180 ^a^	R	RT	R	RT	na	na	R	RT	HS	RI
6	TDr 96/00604 ^a^	R	RT	R	RT	na	na	na	Na	R	RT
7	TDr 99/02674 ^a^	R	RT	R	RT	na	na	R	RT	R	RT
8	TDr 89/02475 ^b^	R	RT	R	RT	na	na	R	RT	R	RT
9	TDr 89/02672 ^a^	R	RT	R	RT	R	RT	R	RT	HS	RI
10	TDr 96/01817 ^a^	R	RT	R	RT	R	RT	R	RT	R	RT
11	TDr 97/00840 ^a^	HS	RI	R	RT	R	RT	R	RT	HS	RI
12	TDr 07/00873 ^a^	R	RT	R	RT	na	na	R	RT	R	RT
13	TDr 02/00515 ^a^	HS	RI	R	RT	R	RT	R	RT	R	RT
14	TDr 00/00403 ^a^	HS	RI	HS	RI	R	RT	R	RT	R	RT
15	TDr 07/00168 ^a^	HS	RI	HS	RI	R	RT	R	RT	R	RT
16	TDr 95/18544 ^a^	HS	RI	HS	RI	HS	RI	R	RT	HS	RI
17	TDr 98/00933 ^a^	HS	RI	HS	RI	R	RT	R	RT	R	RT
18	TDr 01/00405 ^a^	R	RT	HS	RI	na	na	R	RT	R	RT
19	TDr 03/00058 ^a^	HS	RI	R	RT	HS	RI	R	RT	HS	RI
20	TDr Dente ^c^	R	RT	R	RT	R	RT	R	RT	R	RT
21	TDr Agbanwobe ^c^	R	RT	R	RT	R	RT	HS	RI	na	na
22	TDr 08-21-2 (Ekpe) ^c^	R	RT	HS	RI	R	RT	R	RT	R	RT
23	TDr Alumako^c^	R	RT	HS	RI	na	na	R	RT	R	RT
24	TDr 2341 (Amula) ^c^	R	RT	HS	RI	R	RT	R	RT	HS	RI
25	TDr Makakusa ^c^	HS	RI	R	RT	R	RT	R	RT	HS	RI
26	TDr Bp122 (TDr Pouna) ^c^	HS	RI	HS	RI	na	na	na	Na	R	RT

*: TDr = tropical *Dioscorea rotundata.* ^a^ = breeder’s lines; ^b^ = an improved variety released in Nigeria; ^c^ = landraces. A: Designation of host response to nematode feeding in an experimental trial after Sasser et al., 1984 [18]: R = resistant; HS = hypersusceptible; B: host designation after Starr et al., 2013 [14]; RT = resistant and tolerant; RI = resistant but intolerant; na = insufficient planting material.

**Table 2:** Reaction of vine cuttings of used reference genotypes and observation in other studies using tubers as planting materials.

N	Accession	*M. incognita*			*S. bradys*		
		PS ^α^	OS	Reference	PS ^α^	OS	Reference
8	TDr 89/02475	R	S	Bamkefa, 2010 [21]	R	S	Bamkefa, 2010 [21]
16	TDr 95/18544	HS	HS	Bamkefa, 2010 [21]	HS	HS	Bamkefa, 2010 [21]
24	TDr Amula	R ^β^	S	Bamkefa, 2010 [21]	HS	S	Bamkefa, 2010 [21]
25	TDr Makakusa	HS	S	Kolombia et al., 2017 [9]	HS	S	Kolombia, 2017 [23,24]
26	TDr Pouna	HS	S	Osei et al., 2015 ^γ^ [22]	R	S	Bamkefa, 2010 [21]

N: corresponding genotype serial number on Table 1. ^α^: PS = observation in the present study, OS = observation in the referred study. ^β^: TDr (Amula) is hypersusceptible to *M. javanica* in the present study. ^γ^: observation based on natural infection from field, host reaction: R = resistant, HS = hypersusceptible, S = susceptible. Reactions in bold refer to similar reactions in the PS and OS.

**Table 3:** Performance from vine cuttings and semi-autotrophic hydroponics (SAHs) (tuber and root weight).

Propagation Technique	Plant Parameters	Tuber Weight (g) ^α^			Root Weight (g)		
	Yam Accession	Control	Mi	Mj	Control	Mi	Mj
*	TDr 95/19177SAH	16.2	15.4	11.5	4	2.1	4.3
	SAHs	1.8 a	2.5 a	0.9 a	5.5 a	2 a	3.1 a
TDr Makakusa	VC	0.2 b	0.2 b	0.3 a	0.04 b	0.1 a	0.2 b
	*p*	0.0109	0.007	0.0765	0.0027	0.0838	0.0198

* SAHs = semi-autotrophic hydroponics; VC = vine cutting. ^α^ Means are values of five replicates (SAHs) and seven replicates (VC). Means with the same letter within a column are not significantly different at *p* ≤ 0.05.

**Table 4:** Nematode (*Meloidogyne incognita* and *M. javanica*) performance and plant reactions from vine cuttings (VCs) and semi-autotrophic hydroponics (SAHs).

Propagation Technique	Nematode Parameters Yam Accession	Damage Index			RF *		Reaction *	
		Control	Mi *	Mj *	Mi *	Mj *	Mi *	Mj *
	TDr 95/19177SAH *	1	4	3.8	6.8	6.9	S	S
	SAHs	1	4.2 a	3.2 a	4 a	9.6 a	S	S
	TDr MakakusaVC *	1	2.3 b	2.4 a	0.005 b	0.039 a	HS	R
	*p*	-	0.0014	0.5028	0.0002	0.0655		

* SAHs = semi-autotrophic hydroponics; VC = vine cutting. Mi = *Meloidogyne incognita*; Mj = *M. javanica*; RF = reproduction factor; reaction = plant reaction to nematodes; S = susceptible; HS = hypersusceptible; R = resistant. Means with the same letter within a column are not significantly different at *p* ≤ 0.05.

**Table 5:** Host designation using damage index and reproduction factor: Insights from Sasser et al., 1984 [18] and Starr et al., 2013 [14].

Host Efficiency “HEf” (Susceptible–Resistant)	Host Sensitivity “HSe” (Tolerant–Intolerant)	Sasser et al., 1984 [18]	Starr et al., 2013 [14]
Reproduction Factor (Rf)	Plant Damage * (Gall Index)	Class	Class
≤1 R	≤2 T	1-R	1-RT
≤1 R	>2 I	2-HS	2-RI
>1 S	≤2 T	3-T	3-ST
>1 S	>2 I	4-S	4-SI

* Plant damage: plant damage index (DI); R = resistant, S = susceptible, T = tolerant, I = intolerant, HS = hypersusceptible.

## Data Availability

The data presented in this study are available on request from the corresponding author.

## Supplementary Materials

The following supporting information can be downloaded at: https://www.mdpi.com/article/10.3390/plants13091175/s1, Figure S1. (A) Yam vine cuttings survival rate (%) of 17 Breeeder’s and one released yam genotype four weeks after planting (WAP). (B) Yam vine cuttings survival rate (%) of seven landraces 4 WAP. Figure S2. Yam semi-autotrophic hydroponics (SAH) survival rate (%) of two selected genotypes Tdr-Makakusa and Tdr95/19177 five weeks after planting (WAP). Figure S3. Tubers of variable sizes harvested from yam vine cuttings survival rate 12 weeks after transplantation. Scale bar: 1 unit = 1 cm. Table S1. Tuber weight, galling index (GI), nematode density in roots and rating of accessions inoculated with *Meloidogyne incognita.* Table S2. Tuber weight, plant galling index (GI), nematode produced and rating of accessions reaction to *Meloidogyne javanica.* Table S3. Tuber weight, plant galling index (GI), nematode produced and rating of accessions reaction to *Meloidogyne enterolobii.* Table S4. Tuber weight, plant galling index (GI), nematode produced and rating of accessions reaction to *Meloidogyne arenaria.* Table S5. Tuber weight, plant damage index (DI), nematode produced and rating of accessions reaction to *Scutellonema bradys.*
