# Supplementary material for: An Improved Phenotyping Method for Evaluation of Yam (Dioscorea spp.) Resistance to Nematodes Belonging to the Genera Meloidogyne and Scutellonema
Source: Plants (Basel). 2024 Apr 23;13(9):1175. doi: 10.3390/plants13091175 (PMC11085691; doi:10.3390/plants13091175)
Supplement: Supplementary file 1 [file plants-13-01175-s001.zip › plants-2920536-supplementary.pdf]

plants

Article

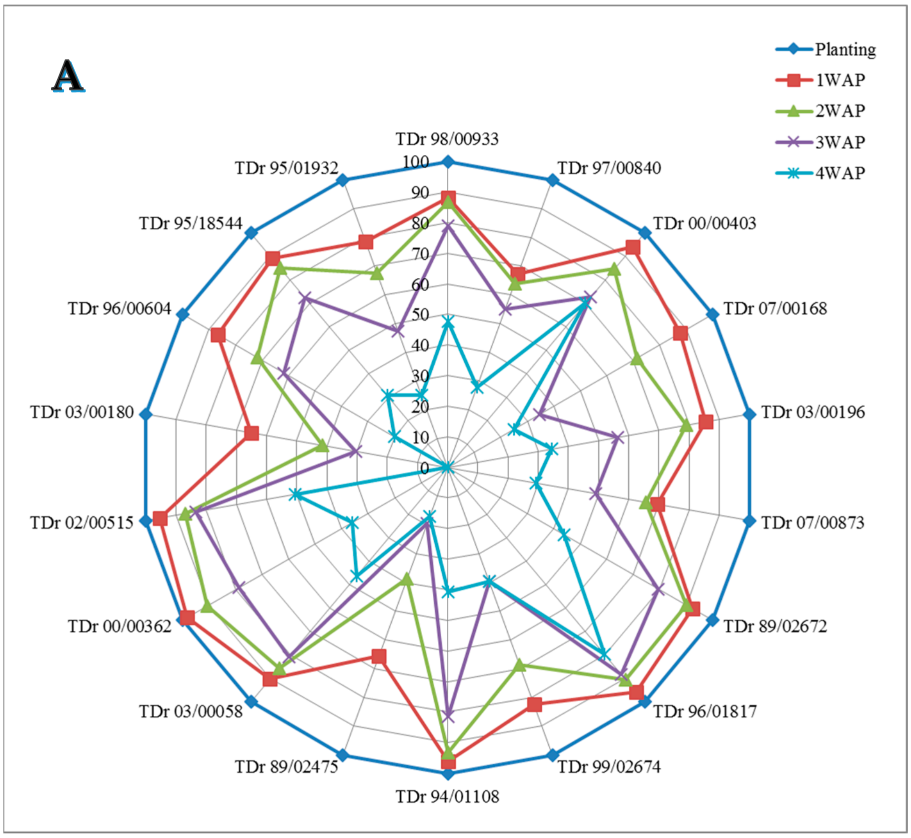

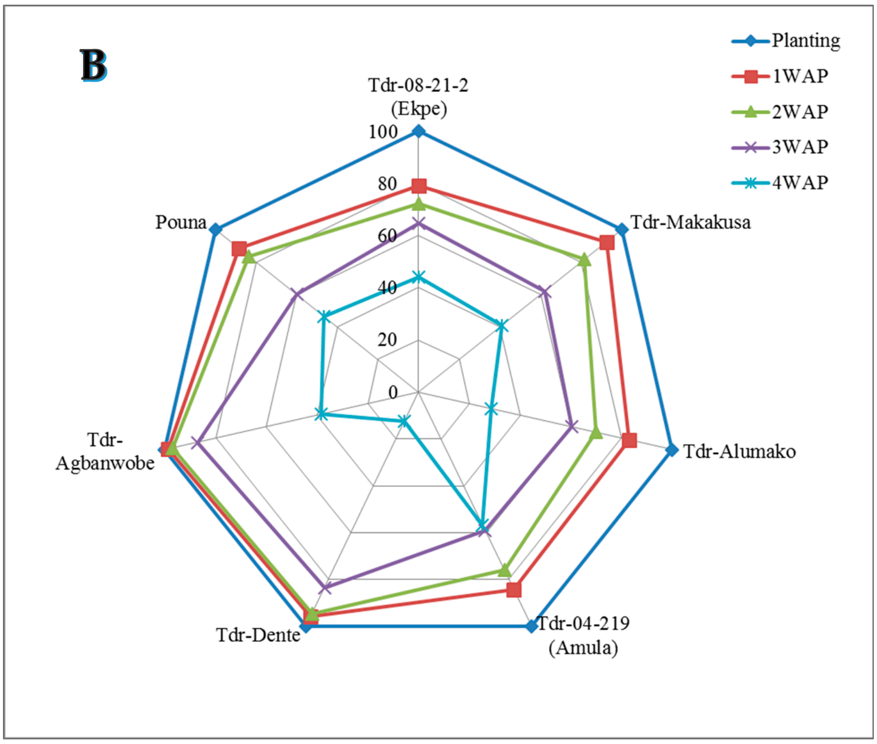

Supplementary Figure S1. (A) Yam vine cuttings survival rate (%) of 17 Breeder's and one released yam genotype four weeks after planting (WAP). (B) Yam vine cuttings survival rate (%) of seven landraces 4 WAP.

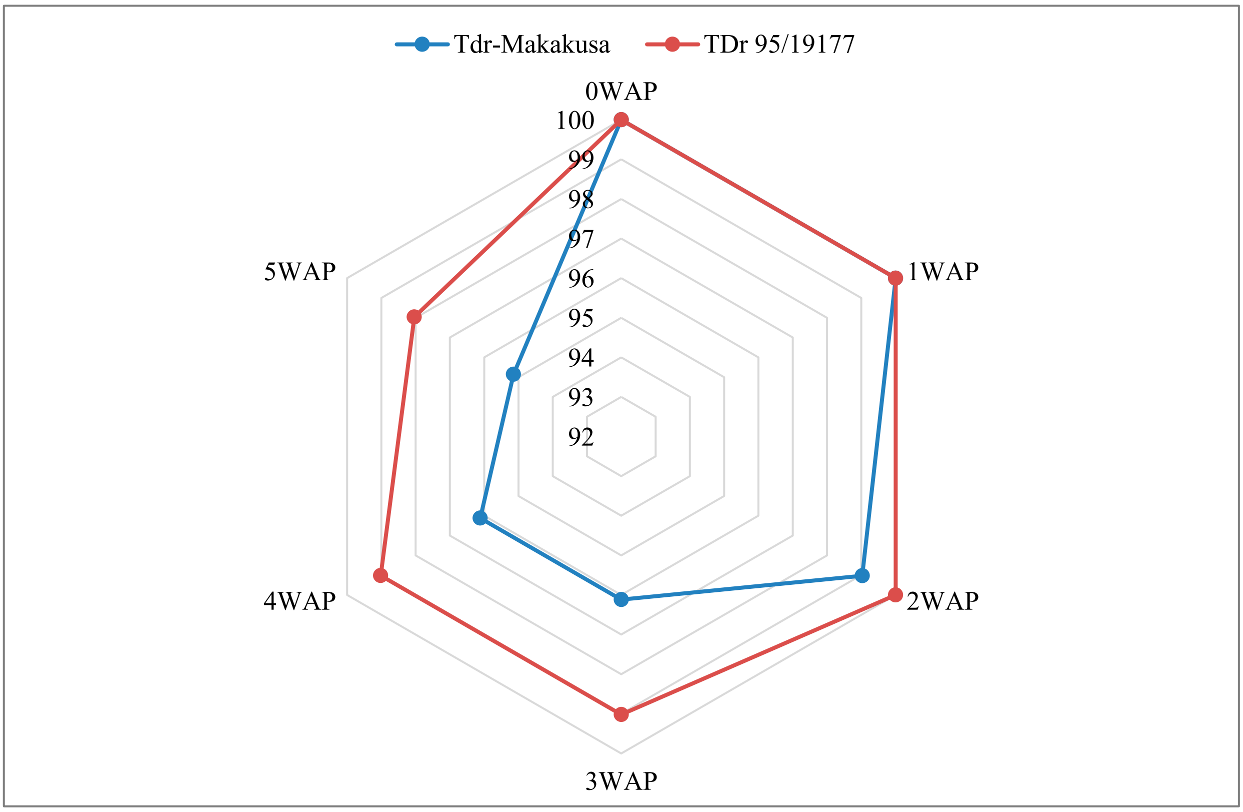

Supplementary Figure S2. Yam semi-autotrophic hydroponics (SAH) survival rate (%) of two selected genotypes Tdr-Makakusa and Tdr95/19177 five weeks after planting (WAP).

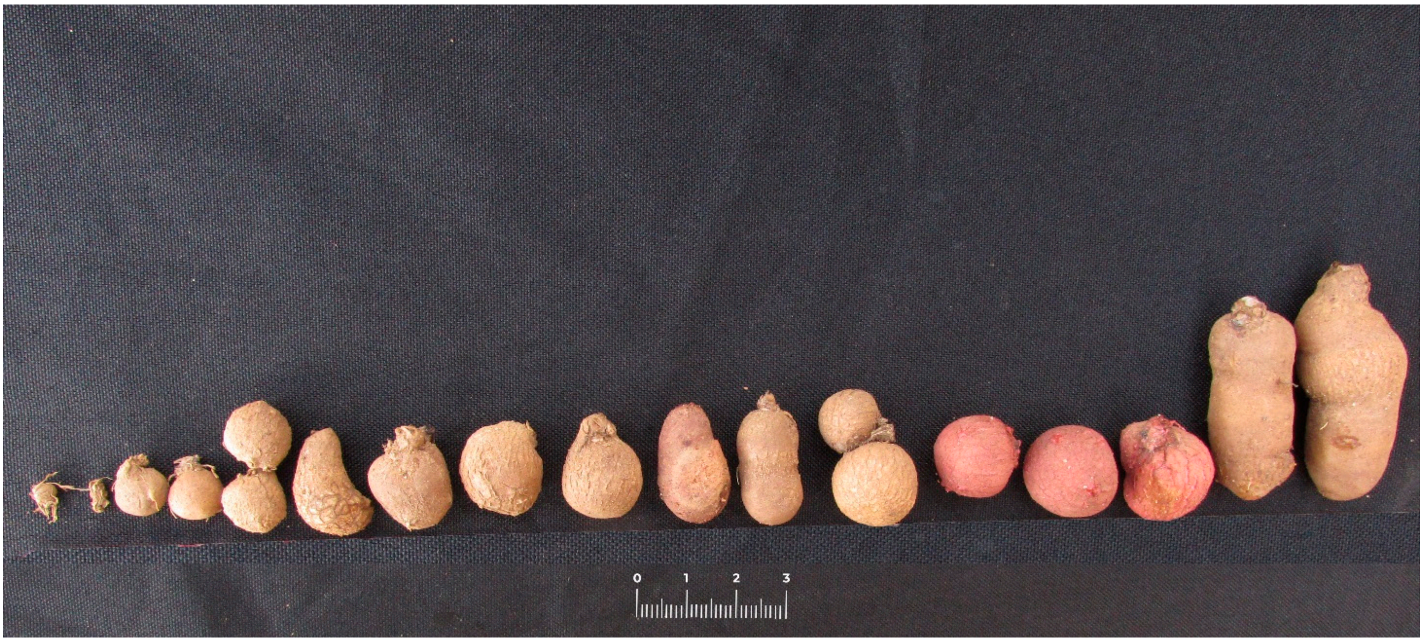

Supplementary Figure S3. Tubers of variable sizes harvested from yam vine cuttings survival rate 12 weeks after transplantation**.** Scale bar: 1 unit=1 cm

Supplementary Table S1. Tuber weight, galling index (GI), nematode density in roots and rating of accessions inoculated with *Meloidogyne incognita.*

Type	No	Genotype	Tuber weight [α] (g) ± SE		Nematode g⁻¹ root	GI	Rating
			Control	*M. incognita*	Mean (Min-Max)		
	1	TDr 03/00196	0	0	66.7 (0 -200)	2	R
	2	TDr 00/00362	0.5 B ± 0	0.6 B ± 0.1	13.3 (0 -40)	1.5	R
	3	TDr 95/01932	0.3 B	0.4 B ± 0.2	0	1.8	R
	4	TDr 94/01108	0.2 B ± 0.1	0.5 B ± 0.4	0	1.8	R
	5	TDr 03/00180	0.8 B ± 0.3	0.6 B ± 0.1	0	1.6	R
	6	TDr 96/00604	0.1 B	0.4 B ± 0.1	0	1.7	R
	7	TDr 99/02674	0.1 B ± 0.1	0.2 B ± 0	4.7 (0 -19)	2	R
	8	TDr 89/02475	0.2 B ± 0	0.6 B ± 0.2	0	1.3	R
**Breeeder's**	9	TDr 89/02672	0.7 B ± 0.2	0.8 B ± 0.2	23.8 (0 -167)	2.2	HS
**lines**	10	TDr 96/01817	1.0 B ± 0.4	1.6 B ± 0.8	47.4 (0 -140)	2.6	HS
	11	TDr 97/00840	0.4 B ± 0.2	0.3 B ± 0.1	38.1 (0 -167)	2.4	HS
	12	TDr 07/00873	.	0.1 B	0	1.8	R
	13	TDr 02/00515	0.6 B ± 0.4	0.8 B ± 0.4	11.4 (0 -58)	2.6	HS
	14	TDr 00/00403	0.3 B ± 0.2	0.5 B ± 0.2	50 (0 -200)	2.2	HS
	15	TDr 07/00168	1.0 B	0.5 B ± 0.1	11.1 (0 -50)	2.6	HS
	16	TDr 95/18544	1.0 B ± 0.3	0.8 B ± 0.2	601.5 (0 -1800)	2.6	HS
	17	TDr 98/00933	0.4 B ± 0.1	0.7 B ± 0.3	131.8 (0 -903)	2.7	HS
	18	TDr 01/00405	3.2 A ± 0.7	2.5 A ± 1.0	0	2.5	HS
	19	TDr 03/00058	2.0 B ± 0.4	1.6 B ± 0.4	43.8 (0 -243)	3	HS
		**P**	<.0001	0.0426	0.2638		
	20	TDr Dente	0.2 A ± 0.1	0.1 B ± 0	0	1.3	R
	21	TDr Agbanwobe	0.4 A ± 0.2	0.4 B ± 0.2	75 A (0 -150)	2	R
	22	TDr 08-21-2 (Ekpe)	0.8 A ± 0.3	1.5 A ± 0.3	49.5 A (0 -170)	1.9	R
**Landrace**	23	TDr Alumako	0.5 A ± 0.2	0.2 B ± 0.1	0	1	R
	24	TDr 04-219 (Amula)	0.9 A ± 0.2	0.8 B ± 0.2	11.4 A (0 -58)	1.4	R
	25	TDr Makakusa	0.2 A ± 0.1	0.2 B ± 0.1	288.6 A (0 -1700)	2.3	HS
	26	TDr Pouna	0.8 A ± 0.3	0.1 B ± 0	100 A (0 -400)	2.4	HS
		**P**	**0.3807**	**0.0002**	**0.6922**		

SE= Standard Error, **GI**= galling index, **Rating**=Host reaction rating (**R**=Resistant, **HS**=Hyper susceptible, **na**=not applicable due to shortage of planting material), Galling score: 1-5 where 1 represents 0 galls and 5 represents >30 galls per root system. Tubers weights with the same letter in the same column are not significantly different according to the SNK test ($P \leq 0.05$); when no significant different no letter is assigned. **Nematode g^{-1}** = number of nematodes per gram of root, [α] Means are values of seven replicates.

Supplementary Table S2. Tuber weight, plant galling index (GI), nematode produced and rating of accessions reaction to *Meloidogyne javanica.*

Type	No	Genotype	Tuber Weight (g) ± SE		Nematode g-1 root	GI	Rating
			Control	M. javanica	Mean (Min-Max)		
Breeeder's lines	1	TDr 03/00196	0	0.2 B ± 0.1	0	1.5	R
	2	TDr 00/00362	0.5 B ± 0	0.5 B ± 0.1	0	1.8	R
	3	TDr 95/01932	0.3 B	0.1 B	0	1	R
	4	TDr 94/01108	0.2 B ± 0.1	0.5 B ± 0.1	11.8 (0 -40)	1.8	R
	5	TDr 03/00180	0.8 B ± 0.3	0.6 B ± 0.4	21.4 (0 -65)	1.7	R
	6	TDr 96/00604	B ± 0.19.9	2.4 AB ± 1.3	0	1.8	R
	7	TDr 99/02674	0.1 B ± 0.1	0.3 B ± 0.1	0	1	R
	8	TDr 89/02475	0.2 B ± 0	0.3 B ± 0.1	0	1.4	R
	9	TDr 89/02672	0.7 B ± 0.2	0.9 B ± 0.3	4.2 (0 -25)	1.4	R
	10	TDr 96/01817	1.0 B ± 0.4	1.6 AB ± 0.4	6.7 (0 -28)	1.9	R
	11	TDr 97/00840	0.4 B ± 0.2	1.0 B ± 0.2	38.6 (0 -146)	1.7	R
	12	TDr 07/00873	.	.	126.4 (0 -317)	1	R
	13	TDr 02/00515	0.6 B ± 0.4	0.4 B ± 0.1	0	1.7	R
	14	TDr 00/00403	0.3 B ± 0.2	0.6 B ± 0.4	8.9 (0 -45)	2.3	HS
	15	TDr 07/00168	1.0 B	0.3 B ± 0.1	42.2 (0 -145)	2.4	HS
	16	TDr 95/18544	1.0 B ± 0.3	0.7 B ± 0.1	14.2 (0 -75)	2.3	HS
	17	TDr 98/00933	0.4 B ± 0.1	0.5 B ± 0.2	17.1 (0 -72)	2.4	HS
	18	TDr 01/00405	3.2 B ± 0.7	3.2 A ± 1.3	27.7 (0 -139)	3	HS
	19	TDr 03/00058	2.0 B ± 0.4	1.0 B ± 0.3	23.7 (0 -75)	2.1	HS
		**P**	**<.0001**	**0.0001**	**0.1631**		
Land-race	20	TDr Dente	0.2 ± 0.1	0.1 B	0	2	R
	21	TDr Agbanwobe	0.4 ± 0.2	0.3 B ± 0.1	0	1.8	R
	22	TDr 08-21-2 (Ekpe)	080.3	1.9 A ± 0.2	168.4 AB (0 -900)	2.4	HS
	23	TDr Alumako	0.5 ± 0.2	0.8 B ± 0.2	3.1 A (0 -12)	2.7	HS
	24	TDr 04-219 (Amula)	0.9 ± 0.2	0.4 B ± 0.1	30.2 AB (0 -70)	2.3	HS
	25	TDr Makakusa	0.2 ± 0.1	0.3 B ± 0.1	23.8 A (0 -119)	2.4	HS
	26	TDr Pouna	0.8 ± 0.3	0.3 B ± 0.1	991.3 A (158 - 2367)	3	HS
		**P**	**0.3807**	**<.0001**	**0.0052**		

SE= Standard Error, **GI**= galling index, **Rating**=Host reaction rating (**R**=Resistant, **HS**=Hyper susceptible, **na**=not applicable due to shortage of planting material), Galling score: 1-5 where 1 represents 0 galls and 5 represents >30 galls per root system. Tubers weights with the same letter in the same column are not significantly different according to the SNK test (P ≤ 0.05); when no significant different no letter is assigned. **Nematode g^{-1}** = number of nematodes per gram of root, $^{\alpha}$ Means are values of seven replicates.

Supplementary Table S3. Tuber weight, plant galling index (GI), nematode produced and rating of accessions reaction to *Meloidogyne enterolobii.*

**Type**	**No**	**Genotype**	**Tuber Weight (g) ± SE**		**Nematode g^{-1} root**	**GI**	**Rating**
			**Control**	***M. enterolobii***	**Mean (Min-Max)**		
	1	TDr 03/00196	0	0.0 A	20 A (0 -40)	2	R
	2	TDr 00/00362	0.5 B ± 0	.	.	1	R
	3	TDr 95/01932	0.3 B	0.2 A ± 0	0	1	R
	4	TDr 94/01108	0.2 B ± 0.1	1.1 A ± 0.2	2.6 A (0 -10)	2.9	HS
	5	TDr 03/00180	0.8 B ± 0.3		.		Na
	6	TDr 96/00604	0.1 B		.		Na
	7	TDr 99/02674	0.1 B ± 0.1		.		Na
	8	TDr 89/02475	0.2 B ± 0		.		Na
**Breeder's lines**	9	TDr 89/02672	0.7 B ± 0.2	1.5 A ± 0.9	0	1.8	R
	10	TDr 96/01817	1.0 B ± 0.4	2.4 A ± 0.8	0	1.8	R
	11	TDr 97/00840	0.4 B ± 0.2	0.6 A ± 0.3	0	1	R
	12	TDr 07/00873	.		.		Na
	13	TDr 02/00515	0.6 B ± 0.4	0.8 A ± 0.7	0	1	R
	14	TDr 00/00403	0.3 B ± 0.2	0.3 A ± 0.1	0	1.6	R
	15	TDr 07/00168	1.0 B	0.2 A ± 0.2	0	1.7	R
	16	TDr 95/18544	1.0 B ± 0.3	1.5 A ± 0.8	181.9 A (0 -1106)	3	HS
	17	TDr 98/00933	0. B ± 40.1	0.7 A ± 0.3	159.9 A (0 -1000)	2.3	HS
	18	TDr 01/00405	3.2 A ± 0.7		.		Na
	19	TDr 03/00058	2.0 B ± 0.4	2.9 A ± 1.2	17.1 A (0 -69)	2.8	HS
		**P**	**<.0001**	**0.2460**	**0.2787**		
	20	TDr Dente	0.2 A ± 0.1	.	.	1	R
	21	TDr Agbanwobe	0.4 A ± 0.2	0.2 B ± 0	0	2	R
	22	TDr 08-21-2 (Ekpe)	0.8 A ± 0.3	0.8 A ± 0.2	46.7 A (0 -187)	2.2	HS
**Landrace**	23	TDr Alumako	0.5 A ± 0.2		.		Na
	24	TDr 04-219 (Amula)	0.9 A ± 0.2	0.2 B ± 0	94.4 A (0 -567)	2.2	HS
	25	TDr Makakusa	0.2 A ± 0.1	0.1 B	0	2	R
	26	TDr Pouna	0.8 A ± 0.3		.		Na
		**P**	**0.3807**	**0.0212**	**0.8627**		

SE= Standard Error, **GI**= galling index, **Rating**=Host reaction rating (**R**=Resistant, **HS**=Hyper susceptible, **na**=not applicable due to shortage of planting material), Galling score: 1-5 where 1 represents 0 galls and 5 represents >30 galls per root system. Tubers weights with the same letter in the same column are not significantly different according to the SNK test (P ≤ 0.05); when no significant different no letter is assigned. **Nematode g^{-1}** = number of nematodes per gram of root, $^{\alpha}$ Means are values of seven replicates.

Supplementary Table S4. Tuber weight, plant galling index (GI), nematode produced and rating of accessions reaction to *Meloidogyne arenaria.*

Type	No	Genotype	Tuber Weight (g) ± SE		Nematode g^{-1} root	GI	Rating
			Control	*M. arenaria*	Mean (Min-Max)		
Breeder's lines	1	TDr 03/00196	0	0.1 B ± 0.1	35.3 A (0 -100)	1	R
	2	TDr 00/00362	0.5 B ± 0	0.2 B ± 0.1	50 A (0 -100)	2	R
	3	TDr 95/01932	0.3 B	0.2 B ± 0.1	0	2	R
	4	TDr 94/01108	0.2 B ± 0.1	0.2 B ± 0.1	8.9 A (0 -45)	2	R
	5	TDr 03/00180	0.8 B ± 0.3	.	0	1	R
	6	TDr 96/00604	0.1 B		.		Na
	7	TDr 99/02674	0.1 B ± 0.1	0.2 B ± 0.1	0	2	R
	8	TDr 89/02475	0.2 B ± 0	0.1 B ± 0.1	0	1.3	R
	9	TDr 89/02672	0.7 B ± 0.2	0.7 B ± 0.4	66.7 A (0 -140)	1.3	R
	10	TDr 96/01817	1.0 B ± 0.4	2.8 A ± 0.9	2.9 A (0 -21)	1.6	R
	11	TDr 97/00840	0.4 B ± 0.2	0.3 B ± 0.3	80 A (80 -80)	1.7	R
	12	TDr 07/00873	.	.	.	1	R
	13	TDr 02/00515	0.6 B ± 0.4	0.3 B ± 0	0	1.6	R
	14	TDr 00/00403	0.3 B ± 0.2	0.3 B ± 0.1	99 A (0 -320)	2	R
	15	TDr 07/00168	1.0 B	0.5 B ± 0.1	0	1.8	R
	16	TDr 95/18544	1.0 B ± 0.3	0.4 B ± 0	0	1.2	R
	17	TDr 98/00933	0.4 B ± 0.1	0.1 B ± 0	0	2	R
	18	TDr 01/00405	3.2 B ± 0.7	1.6 B ± 0.5	0	2	R
	19	TDr 03/00058	2.0 B ± 0.4	0.4 B ± 0.1	310 A (0 -1860)	1	R
		**P**	**<.0001**	**0.0015**	**01807**		
Landrace	20	TDr Dente	0.2 A ± 0.1	.	.	1	R
	21	TDr Agbanwobe	0.4 A ± 0.2	0.1 A ± 0.1	166.7 A (0 -334)	2	R
	22	TDr 08-21-2 (Ekpe)	0.8 A ± 0.3	1.0 A ± 0.4	3.3 A (0 -17)	1.2	R
	23	TDr Alumako	0.5 A ± 0.2	.	0	1.7	R
	24	TDr 04-219 (Amula)	0.9 A ± 0.2	0.7 A ± 0.1	102.5 A (0 -410)	2.3	HS
	25	TDr Makakusa	0.2 A ± 0.1	0.2 A ± 0.1	18.8 A (0 -57)	1.7	R
	26	TDr Pouna	0.8 A ± 0.3		.		Na
		**P**	**0.3807**	**0.1662**	**0.8109**		

SE= Standard Error, **GI**= galling index, **Rating**=Host reaction rating (**R**=Resistant, **HS**=Hyper susceptible, **na**=not applicable due to shortage of planting material), Galling score: 1-5 where 1 represents 0 galls and 5 represents >30 galls per root system. Tubers weights with the same letter in the same column are not significantly different according to the SNK test (P ≤ 0.05); when no significant different no letter is assigned. **Nematode g^{-1} =** number of nematodes per gram of root, $^{\alpha}$ Means are values of seven replicates.

Supplementary Table S5. Tuber weight, plant damage index (DI), nematode produced and rating of accessions reaction to *Scutellonema bradys.*

Type	No	Genotype	Tuber Weight (g) A ± SE		Nematode g^{-1} root	DI	Rating
			Control	*S. bradys*	Mean (Min-Max		
	1	TDr 03/00196	0.0 B ± 0.0	0.1 B ± 0.0	68 A (0 -200)	2	R
	2	TDr 00/00362	0.5 B ± 0.0	0.4 B ± 0.1	708.8 A (16 -2040)	2.3	HS

	3	TDr 95/01932	0.3 B	0.5 B ± 0.1	12.2 A (0 -23)	2.3	HS
	4	TDr 94/01108	0.2 B ± 0.1		.		Na
	5	TDr 03/00180	0.8 B ± 0.3	0.6 B ± 0.1	82.1 A (0 -165)	2.5	HS
	6	TDr 96/00604	0.1 B	.	0	0.3	R
	7	TDr 99/02674	0.1 B ± 0.1	0.2 B ± 0.1	0	1.4	R
	8	TDr 89/02475	0.2 B ± 0.0	0.2 B ± 0.1	0	2	R
**Breeder's lines**	9	TDr 89/02672	0.7 B ± 0.2	2.1 A ± 1.3	78.8 A (0 -148)	3.8	HS
	10	TDr 96/01817	1.0 B ± 0.4	2 B ± 0.5	82.3 A (0 -377)	2.9	HS
	11	TDr 97/00840	0.4 B ± 0.2	0.5 B ± 0.2	55.8 A (0 -260)	3	HS
	12	TDr 07/00873	.	0.1 B ± 0.1	17 A (10 -25)	0.8	R
	13	TDr 02/00515	0.6 B ± 0.4	0.5 B ± 0.1	0	1	R
	14	TDr 00/00403	0.3 B ± 0.2	0.4 B ± 0.1	36.9 A (0 -112)	2.4	HS
	15	TDr 07/00168	1.0 B	0.5 B	0	2	R
	16	TDr 95/18544	1.0 B ± 0.3	1 B ± 0.3	55 A (0 -110)	3	HS
	17	TDr 98/00933	0.4 B ± 0.1	0.5 B ± 0.3	13.9 A (0 -50)	1.9	R
	18	TDr 01/00405	3.2 A ± 0.7	1.6 B ± 0.6	0	2	R
	19	TDr 03/00058	2.0 B ± 0.4	0.7 B ± 0.3	9.8 A (0 -30)	3	HS
		**P**	**<.0001**	**0.0104**	**0.0953**		
	20	TDr Dente	0.2 A ± 0.1	0.3 A ± 0.0	20 A (0 -40)	1	R
	21	TDr Agbanwobe	0.4 A ± 0.2		.		Na
	22	TDr 08-21-2 (Ekpe)	0.8 A ± 0.3	1.6 A ± 0.6	112.4 A (18 -354)	2.8	HS
**Landrace line**	23	TDr Alumako	0.5 A ± 0.2	0.5 A ± 0.1	45.4 A (0 -150)	1.7	R
	24	TDr 04-219 (Am-ula)	0.9 A ± 0.2	0.3 A ± 0.1	12 A (0 -60)	2.8	HS
	25	TDr Makakusa	0.2 A ± 0.1	0.2 A ± 0.0	257.7 A (0 -516)	2.5	HS
	26	TDr Pouna	0.8 A ± 0.3	0.2 A	1100 A (900 - 1300)	0.8	R
		**P**	**0.3807**		**0.0719**		

SE= Standard Error, **DI**= damage index, **Rating**=Host reaction rating (**R**=Resistant, **HS**=Hyper susceptible, **na**=not applicable due to shortage of planting material), Damage score scale: 1-5 where 1 represents 0% and 5 represents >75%. Tubers weights with the same letter in the same column are not significantly different according to the SNK test (P ≤ 0.05); when no significant different no letter is assigned. **Nematode. g^{-1} =** number of nematodes per gram of root, $^{\alpha}$ Means are values of seven replicates.
